# Dermal and Transdermal Drug Delivery through Vesicles and Particles: Preparation and Applications

**DOI:** 10.34172/apb.2022.006

**Published:** 2021-02-06

**Authors:** Unnati Garg, Karuna Jain

**Affiliations:** ^1^Amity Institute of Pharmacy, Amity University, Sector-125, Noida, Uttar Pradesh-201303.

**Keywords:** Transdermal, Dermal, Vesicle systems, Particle systems, Niosomes, Ethosomes, Nanoparticles, Transferosomes, Drug delivery

## Abstract

Transdermal delivery over the past decade has become the field of interest for drug delivery due to its various advantages such as no first-pass metabolism, increased drug bioavailability, and easy administration. Different vesicle systems like ethosomes, liposomes, niosomes, and transferosomes along with particle systems like lipid nanoparticles, polymeric nanoparticles, carbon nanotubes, and fullerenes have been developed. These vesicles and particle systems have been developed using various easy and effective methods like cold injection method, rotary film evaporation, thin film hydration, high shear homogenization, solvent extraction method, and many more. These drug delivery systems are a very effective and feasible option for transdermal drug delivery and further developments can be made to increase their use. This article explains in detail the preparation methods and applications for these drug delivery systems.

## Introduction

The skin covers approximately 1.7 m^2^ of the area and is the largest organ of the body. It provides the body a barrier for protection against chemicals, microorganisms, ultraviolet radiation, and prevents water loss from the body.^1^ The skin is composed of different layers namely: the epidermis, dermis, and hypodermis.

Dermal and transdermal routes for drug delivery have gained much importance during the last decade due to their various advantages which include: physicochemical protection for different drugs; improved patient compliance; appropriate for unconscious patients or those who are vomiting; first-pass metabolism is avoided which enhances the bioavailability of the drug; the frequency of dose administration is reduced; and less risk of toxic side effects.^2^

**Figure 1:**
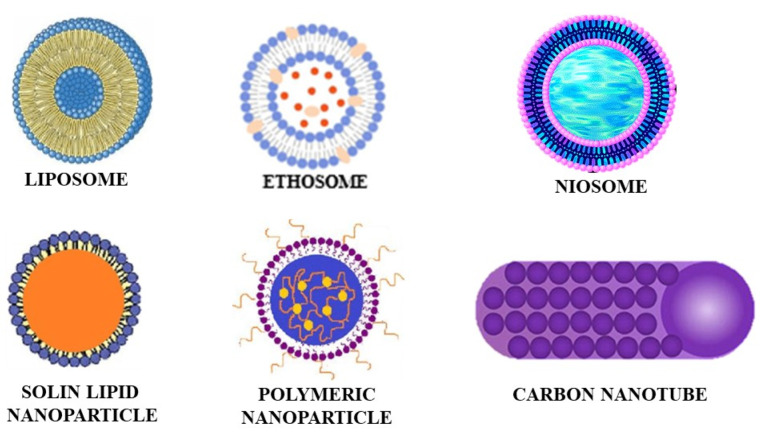
Diagrammatic representation of vesicle and particle systems.

Different vesicle and particle systems have been developed for improved transdermal drug delivery. Vesicle systems like ethosomes, liposomes, niosomes, and transferosomes have been developed. Ethosomes are composed of phospholipids and ethanol; liposomes contain phospholipids and cholesterol; transfersomes are made of surfactants, phospholipids, and water; and niosomes are composed of non-ionic surfactants along with other additives. Particle systems like lipid nanoparticles, polymeric nanoparticles, carbon nanotubes, and fullerenes are used. Lipid nanoparticles are colloidal dispersions and have great stability and tolerability. Polymeric nanoparticles are made from different biocompatible and biodegradable polymers. The carbon nanotubes and fullerenes have carbon as the main component. The carbon nanotubes have a cylindrical structure while the fullerenes are spherical. The diagrammatic representation of these vesicle and particle systems is shown in Figure 1.^3^

The transdermal drug delivery route possesses some limitations along with its several advantages. These include limited efficiency for the delivery of large molecules like proteins; only molecules with quantifiable solubility in both water and oil and well-adjusted lipophilicity can be delivered; and the drug molecules having relatively good pharmacological potency are good candidates for this mode of delivery.^4^

## Dermal and transdermal drug penetration pathways

Drug penetration through the skin follows the transepidermal pathway or appendageal pathway.

The transepidermal pathway involves penetration of the molecule through the intra/or extracellular spaces of the epidermis, dermis, and hypodermis. The molecule follows this pathway either transcellularly (through alternate cellular layers and extracellular matrix) or intracellularly (follows the path through the extracellular matrix).

The appendageal pathway includes penetration either through the sweat ducts or through the hair follicles.^5^

## General preparation methods for the vesicle and particle systems

### Reverse phase evaporation

This method involves the hydration of lipids from the organic solvent directly to obtain an aqueous suspension of multilamellar or unilamellar vesicles. This is a highly efficient method as it allows large-scale entrapment of aqueous material due to the high aqueous space to lipid ratio.

### Solvent injection method

In this method a lipid solution made in diethyl ether is injected in an aqueous solution of the drug to be loaded under reduced pressure or at 55°C to 65°C. when the ether is removed under vacuum, it results in the formation of vesicles.

### Double emulsion technique

This method involves dissolving a drug molecule in an aqueous phase and then emulsifying it with the oil phase to form a primary emulsion, which in turn is then mixed with another aqueous solution to make a double emulsion. After the double emulsion is formed, the solvent is removed which results in the development of drug loaded vesicles.

## Ethosomes

Ethosomes are non-invasive, specially made vesicular carriers invented by Touitou for efficient drug delivery by topical application on the skin.^1^ They are mainly composed of phospholipids, ethanol (up to 50%), and water. This system is mainly characterized by its simplicity in preparation, efficacy, and safety. These are soft and malleable vesicles which are used for efficient delivery of active drug ingredient into deep skin layers and systemic circulation.^6^ The presence of ethanol in high concentration provides Ethosomes a special ability to penetrate the stratum corneum.^7^

Touitou et al did the characterization of the ethosomal system. Using ^31^ P-nuclear magnetic resonance studies and electron microscopy, they demonstrated the presence of vesicles in the system.^8-10^ The vesicular structure was examined using transmission electron microscopy (TEM) which showed that ethosomes can be unilamellar or multilamellar based on the system composition.^8-11^ The transition temperature of the lipids in ethosomes was measured by differential scanning calorimetry (DSC) and then compared with lipid evolution temperature in liposomes. The results showed lower lipid values for ethosomes which implied their higher degree of fluidity.^8-10^

The vesicular size distribution of different composition systems of ethosomes was also measured which suggested that the size of the vesicles can be varied by making changes in the composition of the system and the size ranges between 30nm to few microns. The higher the lipid concentration, the larger will be the vesicle size whereas higher concentrations of ethanol at the same lipid concentration results in small-sized ethosomes.^8^

The entrapment efficiency (EE) (percentage of drug encapsulated) of ethosomes can be calculated using the formula below:


$$EE\%=\;\frac{Amount\;of\;drug\;entrapped}{Total\;amount\;added}\times 100$$

The ethosomes when compared to liposomes and other dermal and transdermal drug delivery systems, were found to be more effective in drug delivery via the skin in terms of depth as well as quantity.^12^

## Methods of preparation of ethosomes

Different methods can be used for the preparation of ethosomes. These include:

1. Cold injection method
2. Hot injection method
3. Vortex/sonication method
4. Rotary film evaporation

### Cold injection method

**Figure 2:**
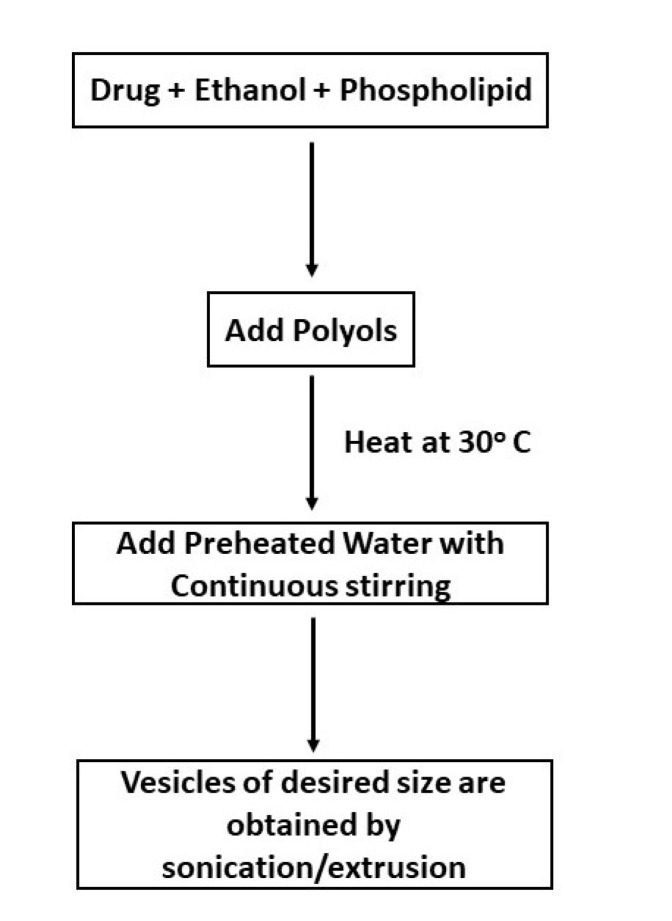
Schematic depiction of Cold Injection Method for preparation of ethosomes.

This technique includes the preparation of a solution of lipids and drug in ethanol to which addition of polyols is done and then heated at 30°C. To this mixture preheated water is added dropwise along with continuous stirring resulting in formation of uniform vesicles. Vesicles of the anticipated size can be attained by sonication, extrusion, etc.^13^ This method is schematically represented in Figure 2.

### Hot injection method

**Figure 3:**
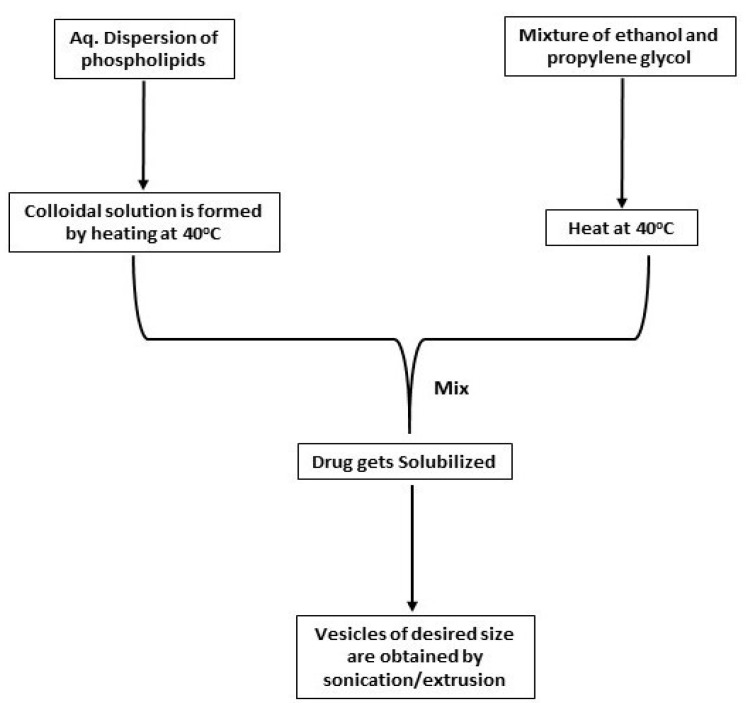
Schematic depiction of hot injection method for preparation of ethosomes.

This technique includes the preparation of a phospholipid aqueous dispersion and heating it at 40°C to form a colloidal solution. The organic solution of propylene glycol and ethanol is then prepared separately and heated at 40°C. After this, both the solutions are mixed, and eventually, the drug gets dissolved in either water or ethanol depending upon its properties. Vesicles of the anticipated size can be attained by sonication, extrusion, etc.^14^ This method is schematically represented in Figure 3.

### Vortex/sonication method

This technique involves mixing of phospholipids and edge activators by forceful shaking and agitation to suspend them in the phosphate buffer. After that sonication of the suspension is done using either a vortex or a bath sonicator. To get vesicles of the anticipated size it can then be passed through membranes of different sizes.^15^

### Rotary film evaporation method

In this technique, dissolution of lipids is done in a round bottom flask containing an organic solvent. The organic solvent is then vaporised using a rotary evaporator resulting in formation of a thin film around the inner walls of the round bottom flask, which is hydrated using aqueous media containing drug which causes swelling of lipids and formation of bilayer vesicles.^16^ Vesicles of the anticipated size can be attained by sonication, extrusion, etc. This method is mainly utilised for developing multilamellar vesicles.^17^

## Applications of ethosomes

Gu et al studied the effect of ethosomes containing 5-fluorouracil in the treatment of laryngotracheal stenosis. They used a rabbit model for the investigation. They concluded that 5-fluorouracil containing ethosomes showed better results than 5-fluorouracil only.^18^

Shen et al developed and evaluated skin deposition and transdermal flux of apigenin encapsulated in ethosomes. They found that skin deposition and transdermal flux of apigenin was improved by encapsulation in ethosomes. They also reduced ultraviolet B light-induced inflammation in mouse skin by causing a reduction of cyclooxygenase 2, thus this delivery system could be used for the treatment of skin inflammation caused by ultraviolet B light.^19^

Goindi et al developed a cetirizine dihydrochloride loaded ethosomes for topical application. They performed permeation studies on mice skin ex-vivo which showed improved skin retention and high permeation flux. From their results, they concluded that ethosomes are potential cetirizine carriers for dermal delivery for the treatment of atopic dermatitis.^20^

Marto et al developed a griseofulvin (an antifungal drug) loaded ethosomal system. They evaluated the permeation of the drug through newborn pigs by using Franz diffusion cells. To check the therapeutic efficacy of the formulation, they also conducted a skin adapted agar diffusion test. They concluded that the developed formulation has the potential to be used for targeting skin dermatophytes.^21^

A marketed product under the brand name Supravir cream is also available which contains the drug Acyclovir loaded in ethosomes for the treatment of herpes.^22^

## Liposomes

Liposomes are vesicles of lipids that are used for carrying the drug across or into the skin. They have been used to deliver antibiotics, anticancer and antifungal agents. The main constituents of liposomes are phospholipid and cholesterol. Phospholipids of different types (phosphoglycerides, sphingolipids) are present along with their hydrolysis products.^23^

The properties of liposomes vary depending upon their size, composition of lipids, method of preparation, and surface charge. Generally, liposomes are spherical in shape and their particle size lies between 30 nm to several microns. They are formed of lipid bilayers that surround the aqueous units.^24^

Liposomes have various advantages which include: increased therapeutic efficacy of the drug; these are non-toxic, biodegradable; flexible and biocompatible; they help in preventing the exposure of sensitive tissues to toxic drugs; they decrease the toxicity of the encapsulated agent.^25^

The EE (percentage of drug encapsulated) of liposomes can be calculated using the formula below:


$$EE\%=\;\frac{Amount\;of\;drug\;entrapped}{Total\;amount\;added}\times 100$$

Along with these advantages, liposomes also have some disadvantages like low half-life, high production cost, low solubility, and sometimes leakage, and fusion of the encapsulated drug/molecules can occur.^25^

## Classification of liposomes

**Figure 4:**
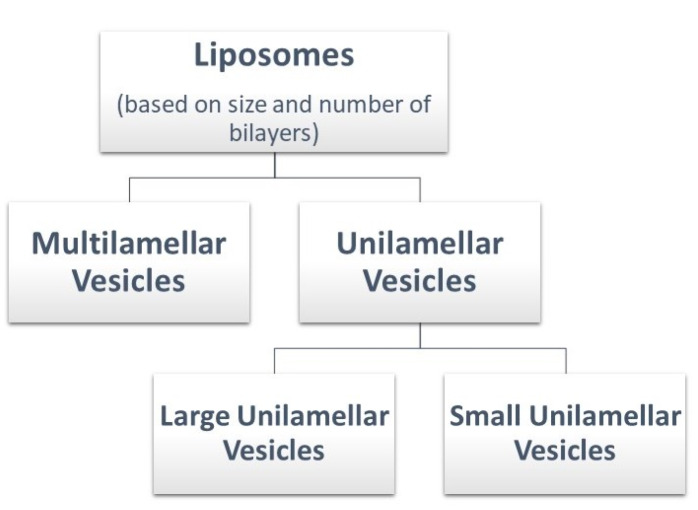
Schematic depiction of the classification of liposomes.

Liposomes can be classified as shown in Figure 4.^26^

## Preparation of liposomes

All the techniques for the preparation of liposomes follow a similar method. The method includes dissolving the lipids in the organic solvent which are then dried and further dispersed in the aqueous media to form liposomes which are then purified.^27^

Different techniques:

1. Thin film hydration
2. Reverse Phase Evaporation and Solvent injection method
3. Detergent depletion method

Thin film hydration: This technique is most widely used. In this method, the lipids are dissolved in an organic solvent, this organic solvent is then vaporised, and the dried lipid film is then dispersed in an aqueous media. The molecule which is to be encapsulated is present either in the aqueous media or in the lipid film.^28^

Reverse phase evaporation:In this method, the lipids are hydrated straight from the organic solvent, and an aqueous suspension of multilamellar or unilamellar vesicles are obtained. This method has a higher efficiency of encapsulation than the film hydration method.^28^

Solvent injection method: In this method a lipid solution made in diethyl ether is injected in an aqueous solution of the drug to be loaded under reduced pressure or at 55°C to 65°C. when the ether is removed under vacuum, it results in the formation of vesicles.

Detergent depletion method: In this method, the lipid film is hydrated with a detergent solution and results in the formation of multilamellar vesicles of large size. This method is time-consuming and shows poor entrapment, therefore it is rarely used.^28^

For industrial-scale manufacturing of liposomes, some other techniques are also used, some of which are: freeze-drying method, heating technique, spray drying method, and supercritical reverse-phase evaporation method.^29^

## Applications of liposomes

Manca et al developed liposomes containing argan oil which were then loaded with allantoin for the treatment of hypertrophic scars and skin ulcers and compared their efficiency with the conventional liposomes. In argan oil containing liposomes, they observed a larger diameter than the conventional liposomes and also the argan oil-containing formulation showed more accumulation of allantoin in the skin and more permeation.^30^

Jose et al developed curcumin-loaded cationic liposomes and made a complex with STAT_3_ siRNA to deal with skin cancer. They performed in-vivo experiments in a mouse model of melanoma skin tumor to check the efficacy of the formulation. This formulation showed improved inhibition of cancer cell growth than either STAT_3_ siRNA only or with curcumin loaded liposomes.^31^

Kapoor et al used liposomes for delivering folic acid through cosmetics and for treating micronutrient deficiencies. They loaded liposomes with folic acid for transdermal delivery which prevented the degradation of folic acid which occurred when given through oral route. They proved that liposomes loaded with folic acid incorporated in cosmetics are feasible for folic acid delivery and showed more stability at room temperature and more transdermal penetration of folic acid. They performed ex-vivo experiments using Franz diffusion cells to check the skin permeation.^32^

Maniyar and Kokare developed lopinavir encapsulated liposomes for use in anti-HIV therapy. They formed these liposomes using the spray drying method. They studied the physicochemical characteristics of the formulation and also performed in-vitro drug release experiments. For the in-vitro experiment, they used cellophane membrane and the results showed enhanced drug release from this formulation than the drug creams.^33^

## Transfersomes

These are vesicular carriers whose basic components include surfactants (edge activators), phospholipids, and water. These are elastic and can be deformed i.e. their shape can be easily changed. Due to this ability, they can easily cross the membrane/channels with a diameter of one-tenth to the vesicles. They squeeze themselves through the channels and can do this without much loss.^34^ Transfersomes get their flexibility due to the surfactants present in them in an optimum ratio.^35^

The advantages and disadvantages of transfersomes are tabulated in Table 1.^34,36^

**Table 1:** Advantages and disadvantages of transfersomes

**Advantages**	**Disadvantages**
Have entrapment efficiency of up to 90% for lipophilic drugs	They can easily undergo oxidative degradation, thus are less stable
Better penetration due to the ability to change shape	These formulations are expensive.
These can be loaded with both high and low molecular weight drugs.	The encapsulation of hydrophobic drugs is difficult.
These can be used for sustained or controlled drug release.	Natural phospholipids might have questionable purity, thus the use of transfersomes can be influenced by it.

Transfersomes can change the composition of their membrane locally and in a reversible fashion depending upon the stress at the site of entry. At the sites where the stress is more, the transfersomes are diluted which causes a decrease in the energetic cost required for deformation of the membrane and allows the particles to pass through the membrane rapidly.^37^

The characterization of the shape and type of vesicles of the transfersomes was carried out using TEM. Dynamic light scattering was used to figure out the size of the vesicles and the size distribution.^38^

The transfersome formulations have a unique and important parameter known as the degree of deformability. For measuring it, the transfersomes are passed through pores of known different sizes in 5 minutes and DLS is used for measuring the size of the particles and their distribution. The following formula was used for calculating the degree of deformability.^38,39^


$$\text{D}=\text{J}\frac{r_{v}}{r_{p}}$$

where,

D: Deformability of vesicle membrane

J: the amount of suspension which was extruded during 5 min

r_v_: the size of vesicles (after passes)

r_p_: pore size of the barrier

The EE (percentage of drug encapsulated) of transfersomes can be calculated using the formula below:


$$EE\%=\;\frac{Amount\;of\;drug\;entrapped}{Total\;amount\;added}\times 100$$

Transfersomes are capable of transferring lipids of about 0.1 mg per hour and cm^2^ area across the skin and this is due to the presence of transdermal osmotic gradients. The transfersomes penetrate the skin along this gradient and it is only possible if the transfersomes can cross the pores on the skin thus they contain surfactants that help them to deform their membrane.^35^

## Preparation of transfersomes

All the methods used for the preparing transfersomes include these 2 basic stages:

1. Preparation of a thin film, its hydration, and then sonication
2. Homogenization of the vesicles obtained by sonication which is done by extrusion through a membrane.^37^

**Figure 5:**
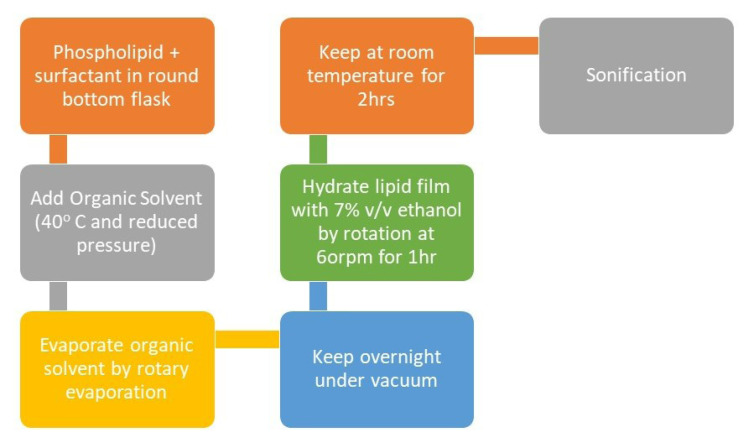
Schematic representation of the preparation of transfersomes.

The conventional method used for the preparation of transfersomes is the Rotary evaporation sonication method as shown in Figure 5. In this method, in a round-bottom flask phospholipid and surfactant are taken and are solubilised in an organic like ethanol. By the use of rotary evaporation, the organic solvent is vaporised under reduced pressure at 40°C. The leftover solvent is then removed by keeping the mixture overnight under vacuum. A lipid-film is deposited at the walls of the flask which is hydrated using 7% v/v ethanol and rotating it at 60rpm for an hour. The formed vesicles were kept at room temperature for 2 hours to swell and turn into large multilamellar vesicles. The size of the vesicles can be reduced by sonication to produce small vesicles.^37,40^

## Applications of transfersomes

Caddeo et al formulated tocopherol acetate encapsulated transfersomes. They studied the morphology of these vesicles and the EE which they found to increase with the increase in the length of the fatty acid. This formulation was efficient in delivering tocopherol to the skin and thus successful in preventing oxidative damage to the skin. Along with the antioxidant action, the formulation also promoted rapid wound closure on the skin.^41^

Wu et al developed resveratrol (antioxidant drug) loaded transfersomes because alone resveratrol is unstable in heat, light, and other conditions. They performed an in-vitro analysis of this formulation and compared it with the results of resveratrol alone. This formulation enhanced skin accumulation by 27.59%. They also performed the cell viability assay which showed that this formulation reduced the cytotoxicity by 34.45%. Thus, they concluded that these resveratrol loaded transfersomes are capable of efficiently delivering the drug.^42^

Fadel et al developed indocyanine green loaded transfersomes to overcome the problem of the high degradation rate of indocyanine green. It is a dye and is used as a photosensitizer in skin diseases. They encapsulated this dye in transfersomes and studied the EE, zeta potential morphology, size of the particles, in-vitro release, and other characteristics of this formulation. This formulation showed sustained release of the drug along with a high clearance rate and minimal pain. Thus, they concluded that this formulation is an effective approach in the treatment of basal cell carcinoma.^43^

Mbah et al formulated NIPRD-AF1(phytomedicine used as antifungal) loaded transfersomes to improve the efficacy of the phytomedicine. They studied the EE and morphology of the vesicles and tested the in-vitro drug release of the formulation using rat skin. From the results, they concluded that the transfersomal formulation was stable and can deliver the drug efficiently.^44^

AL Shuwaili et al developed pentoxifylline loaded transfersomes using sodium cholate. They studied the EE, permeation flux, vesicle diameter, and zeta potential of the formulation. They also checked the drug permeation on rat’s skin and found that the drug permeation was enhanced by 9.1 times. Thus, the authors concluded that this formulation is an effective method for delivering Pentoxifylline.^45^

## Niosomes

Niosomes are vesicular structures like liposomes but instead of phospholipids, they contain non-ionic surfactants as the main component along with some additives. The additives used include cholesterol and some charged molecules. Cholesterol enhances the rigidity of the bilayer. Niosomes prevent the unwanted degradation of the drug molecule in the body.^46-49^

## Method of preparation

The general process for the preparation of niosomes includes the use of a hydration medium for hydrating the non-ionic surfactants. Different methods for noisome preparation are given below:

### Ether injection method

**Figure 6:**
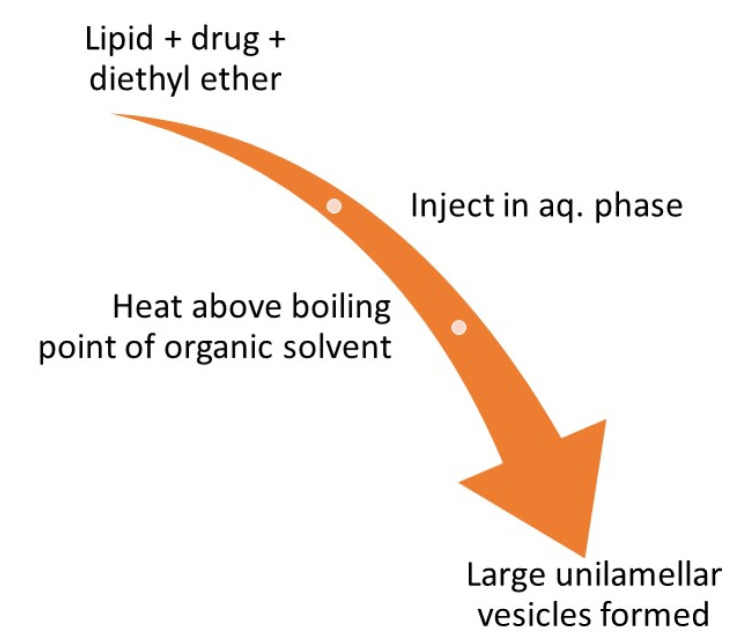
Schematic representation of the preparation of niosomes by ether injection method.

This method involves dissolving the lipid along with the drug in an organic solvent (diethyl ether) and then slowly injecting it in the aqueous medium followed by heating this solution at a temperature more than the boiling point of the organic solvent. This results in the development of big unilamellar vesicles which can be converted to vesicles of desired size.^50^ The diagrammatic representation is shown in Figure 6.

### Reverse phase evaporation method

This technique includes dissolving the surfactants in chloroform and ether mixture and then adding the drug containing aqueous phase to it. The emulsion is formed from this mixture by sonication and then the organic phase is evaporated to form large unilamellar vesicles.^51,52^

### Thin film hydration method

In this method, in round-bottom flask cholesterol and surfactants along with some additives are taken and are solubilised in an organic solvent. Using a rotary evaporator, the organic solvent is evaporated, and a thin film is obtained inside the flask. This film is hydrated using an aqueous medium containing the drug above the transition temperature of the surfactant with continuous shaking. This results in the formation of multilamellar niosomes.^53^

### Microfluidization method

In this process, the drug along with the surfactant solution, under pressure is pumped from a reservoir and then passed through the chamber with packed ice at 100ml/min to cool the solution. During the microfluidization process, the heat produced is removed by passing this solution through a cooling loop. This procedure is continued to obtain the vesicles of the required size.^54^

## Characterization of niosomes

- The characterization of the vesicle size was done using freeze-fracture electron microscopy and TEM.
- Dynamic light scattering, laser light scattering, gel permeation, and gel exclusion methods were used to check the vesicle size distribution.
- The surface charge was measured by free-flow electrophoresis.
- Electric surface potential and surface pH were checked by zeta potential measurements and pH-sensitive probes.
- Small-angle X-ray scattering and P-NMR were used for checking the lamellarity.
- DSC was used to see the phase behavior.
- The EE of the niosomes is also an important factor and it can be measured using the formula given below:

$$EE\%=\;\frac{Amount\;of\;drug\;entrapped}{Total\;amount\;added}\times 100$$

The amount of the entrapped drug can be measured using different methods such as Exhaustive dialysis, centrifugation, ultracentrifugation, and gel filtration.^55^

## Applications of niosomes

Abdelbary and AbouGhaly designed methotrexate (a drug used for the treatment of psoriasis) loaded niosomes for application on the skin to prevent the systemic toxicity caused by methotrexate using the thin film hydration method. They determined the encapsulation efficiency of the formulation which was found to be 78.66% which was very high. They also carried out in-vivo tests and found higher drug deposition than the solution. Also, this formulation was found to be safer than the solution.^56^

Salem et al described tamoxifen citrate (a drug used for the treatment of breast cancer) loaded niosomes to prevent the resistance and toxicity caused by the drug alone using the thin film hydration method. They calculated the EE of the formulation and it was found to be 88.90 ± 0.72%. They incorporated this formulation in chitosan/glyceryl monooleate to form a hydrogel delivery system. After identifying the suitable formula, they evaluated the efficacy of the formulation using the Ehrlich carcinoma mice model. The results showed greater efficacy of this formulation that free tamoxifen citrate. Thus, the authors concluded that these tamoxifen citrate loaded niosomes are a budding drug delivery system for the treatment of breast cancer.^57^

Jacob et al using the coacervation phase separation technique to develop acyclovir loaded niosomes with different concentrations of surfactants, phospholipids, and cholesterol. They performed ex-vivo permeation studies and found that the composition of niosomes influenced the flux values greatly. They evaluated the formulation using the in-vitro and in-vivo rabbit model. From the results, they concluded that this formulation is efficient in delivering acyclovir by topical application.^58^

Pawar et al developed doxorubicin-loaded niosomes and evaluated them. The evaluation showed that the EE was as high as 90%. They also performed in-vivo pharmacokinetic studies which showed improved bioavailability and reduced clearance than the doxorubicin drug solution. Experiments also showed an increased reduction in the tumor by this formulation with reduced toxicity. Thus, the authors concluded that this formulation is efficient in the treatment of cancer.^59^

El-Ridy et al developed lornoxicam-loaded noisome and evaluated them for their anti-inflammatory activity. They used the thin film hydration method for preparing this formulation. They did the characterization of the niosomes formed using DSC, TEM, zeta potential determination, and particle size analysis. They converted these niosomes into a gel using Carbopol 934. They performed ex-vivo studies for skin permeation in Wistar rats and in-vivo studies to check the anti0inflammatory activity of the formulation. The formulation showed EE of up to 66% and high permeation through rat skin. From the results, the authors concluded this lornoxicam-loaded niosomal gel formulation as a potential drug delivery system.^60^

## Lipid nanoparticles

Lipid nanoparticles (LN) are colloidal dispersions which are being used due to their great tolerability, good physical stability, sustained and controlled drug release, increased bioavailability of the drug, and ability to prevent the degradation of drugs. These lipid nanoparticles are of two types: Solid lipid nanoparticles (SLNs) and nanostructured lipid carriers (NLCs).^61,62^

## Solid lipid nanoparticles

These are formed when the lipids in solid form are dispersed in an aqueous medium which is further stabilized with a surfactant. These nanoparticles are good carriers for both lipophilic and hydrophilic drugs but have more preference for lipophilic drugs. The main lipids used to produce these nanoparticles include fatty acids, glycerides, triglycerides, and waxes. Although these SLNs have many advantages but also possess some disadvantages such as uncontrolled drug discharge and poor drug loading capacity.^63^

## Preparation of SLN

### High shear homogenization

**Figure 7:**
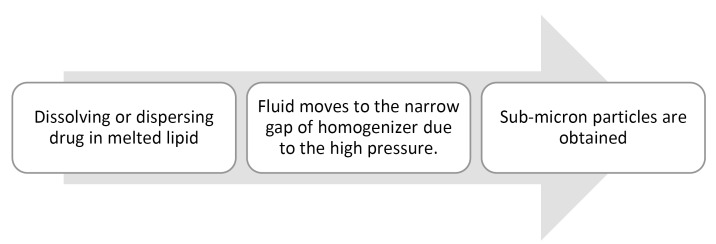
Schematic representation of the preparation of SLNs by High-pressure homogenization.

This method uses dispersion techniques for the preparation of SLNs. This method is potent and can be used for the large scale production of lipid nanoparticles. Hot and cold processes can be used for homogenization. Both the methods include this common procedure shown in Figure 7.^64,65^

In the hot homogenization method, homogenization takes place at temperatures above the lipid melting point, and at high temperatures, the viscosity is reduced which results in smaller particle size.^66^

In the cold homogenization technique, the dissolution of the drug is done in the melted lipids and then the resulting fluid is cooled down rapidly using dry ice or liquid nitrogen. The solid-lipid drug thus obtained is then milled to obtain particles of the desired size (50-100 µm). This powder is then dispersed in an aqueous surfactant solution and this solution is then homogenized at or below room temperature to form SLNs.^67,68^

### Solvent emulsification/evaporation

This technique involves the addition of lipid dissolved in a water-immiscible organic solvent to an aqueous medium containing surfactant to get an emulsion. After this, under reduced pressure, the solvent from the emulsion is evaporated which results in the formation of a dispersion where nanoparticles are dispersed in the aqueous phase.^69^

### Spray drying method

This method is only suitable for the lipids which have melting point above 70^o^C because spray drying involves the use of high temperature and shear forces. It is a cost-effective method and can be used as a substitute for lyophilization.^70^

## Nanostructured lipid carriers

NLCs are the modified version of SLNs which were made to overcome the difficulties in the use of SLNs. In the NLCs the core contains both solid and liquid forms of lipid at an ambient temperature. They form a unique nanostructure that improves the drug encapsulation efficiency and prevents the ejection of the drug during storage.^71,72^

Preparation of NLCs

The general method for the preparation of NLCs includes mixing of solid and liquid lipids which results in the formation of a lipid matrix with a melting point less than that of the solid lipid but stays solid at body temperature.^71^

### The high shear homogenization method

This method of preparation of NLCs is similar to that of SLNs.

### Solvent dispersion method

In this technique, the drug and lipids are dissolved in an organic solvent miscible in water. This solution is added to the aqueous phase comprising of the emulsifier followed by centrifugation to get the NLCs.^73^

### Film-ultrasonic method

In this technique, the drug and the lipids are dissolved in an organic solvent and then the solvent is evaporated using vacuum evaporation leaving a mixed lipid film. To this film, an aqueous solution of a surfactant is added, and then ultrasonic dispersion is done by an ultrasound probe to develop NLCs.^74^

### Ultrasonic emulsion evaporation method

In this technique, the oil phase is formed using the drug and the solid and liquid lipids mixture and this oil phase is then dispersed in the aqueous phase containing surfactant using an ultrasound probe. This solution is cooled and allowed to solidify to form NLCs. After the formation of a stable emulsion, the oil phase is vaporised under reduced pressure.^75^

## Applications of LNs

Gomes et al developed an anti-alopecia compound (minoxidil and finasteride) loaded NLCs to improve the therapy of alopecia. They prepared these lipid nanoparticles using the ultrasonication method and developed particles with an average size of around 200 nm which could efficiently reach the dermis and hair follicle. They found that minoxidil nanoparticles showed loading efficiency as low as 30% while the finasteride nanoparticles showed loading efficiency of up to 90% over 28 days. They performed penetration assays on pig ear skin and found low penetration for both minoxidil and finasteride loaded nanoparticles. With these results, the authors concluded that these formulations have certain good properties which makes them a potent candidate for the dermal delivery of anti-alopecia compounds.^76^

Raza et al developed Tretinoin-loaded NLCs and SLNs and evaluated them. After the preparation of the nanoparticles, these were then incorporated into Carbopol-based hydrogel and then they checked the efficiency of both types of nanoparticles and found that NLCs showed more photoprotection than the SLNs along with high permeation flux. After checking all the results, the authors concluded that these nanoparticles are potent carriers for the treatment of acne and other skin disorders like psoriasis.^77^

Goldinger et al developed Fluconazole-loaded SLNs and NLCs using the solvent diffusion method for the treatment of fungal infections of the skin. They analyzed the antifungal activity of the formulation of immunosuppressed albino rats with induced cutaneous candidiasis. They found the EE of the SLNs and NLCs to be 75.7% ± 4.94 % and 81.4% ± 3.89 % respectively. From the results, they concluded that NLCs are more effective carriers for the transdermal delivery of fluconazole.^78^

## Polymeric nanoparticles

Polymeric nanoparticles have a size ranging from 10-1000 nm and are made from biodegradable and biocompatible polymers. They have a rigid matrix which makes them structurally stable and allows them to keep their structure intact for a longer period after topical application. The use of polymeric nanoparticles enhances the activity of the drug, helps in sustained or controlled release of drugs, and increases drug permeance time in the skin. The drug can be encapsulated in these nanoparticles via different mechanisms namely, entrapment, dissolution, dispersion, or absorption.^79^

Different techniques can be used for the characterization of these nanoparticles: size exclusion chromatography (for determining polymer weight and weight distribution), liquid chromatography (to determine the drug content in the formulation), Dynamic light scattering (to determine the particle size and size distribution), electrophoric mobility (to determine zeta potential and in turn to evaluate the physical stability), small-angle X-ray scattering and DSC (to check the organization of the components of the nanoparticles at a molecular level) and microscopy techniques (to evaluate the surface morphology of the nanoparticles).^80^

## Preparation of polymeric nanoparticles

The basis of the preparation of these polymeric nanoparticles in all the methods is polymerization or precipitation of the pre-formed polymers in-situ.

### Polymerization in emulsion method

In this method, the monomer is added to an aqueous phase containing drug and surfactant, which on heating undergoes polymerization of the monomer in the emulsion and results in the formation of material nanoparticles.^81^

### Interfacial polymerization

In this method, monomer, organic solvent, oil, and drug are added to the aqueous phase containing surfactant. The resulting solution is heated to undergo interfacial polymerization. After polymerization, the organic solvent is evaporated, thus resulting in the formation of vesicular nanostructures.^81^

### Precipitation of pre-formed polymer

In this method, the polymer, solvent, surfactant, drug, and oil (optional) are added to the aqueous phase containing surfactant. This solution is then heated to undergo precipitation. After precipitation has occurred the organic solvent is evaporated and results in the formation of nanoparticles.^81^

### Solvent extraction method

In this method, homogenization of the o/w emulsion formed is done at high speed and then water is added to this solution, and the organic solvent used is evaporated to get the nanoparticles.^81^

## Applications of polymeric nanoparticles

Dong et al investigated dexamethasone-loaded Eudragit L-100 (polymer) nanoparticles for cutaneous drug delivery. The in-vitro analysis of this formulation showed improved drug penetration than the commercial cream formulation. They also evaluated lipophilic drug (Nile Red) loaded nanoparticles which were found to have more transfollicular penetration of the drug. From the results, the authors concluded that the pH-sensitive Eudragit L-100 nanoparticles are a potent candidate for targeted drug delivery of lipophilic drugs.^82^

Hafner et al evaluated melatonin-loaded chitosan/lecithin (polymer) nanoparticles. They determined the drug flux across dermatomed porcine skin and its skin deposition to check the potential of this formulation. This formulation could be used for transdermal delivery of melatonin without causing damage to the plasma membrane at a concentration of up to 200 µg/mL.^83^

A marketed formulation of Minoxidil solution is available which uses poly(lactide-co-glycolide) grafted hyaluronate nanoparticles for the treatment of alopecia.^84^

## Carbon nanotubes and fullerenes

Carbon nanotubes are cylindrical hollow tubes that are made of graphite layers. The number of graphite layers can vary and thus these tubes are classified as Single-walled nanotubes and multi-walled nanotubes.^85^ Due to their hollow structure they can load large volumes of the biomolecules for delivery and their outer structure can be chemically modified to obtain different chemical and physical properties.^71^

These nanotubes have various advantages which make them a suitable candidate for drug delivery:

- They have a hollow structure which makes them suitable for loading biomolecules and also gives them a high surface area to volume ratio.^86^
- They can also be used for gene delivery due to their hydrophobic nature.
- They can be used for targeted drug delivery
- These have great mechanical strength which makes cell adhesion and proliferation easier.^87^

Although these carbon nanotubes possess various advantages, reports have shown that these are known to cause skin irritation after topical application.^88^

Carbon nanotubes are not intended to enter inside the organism and are applied on the skin and only the drug molecules cross the body barriers, thus patches are used which are a self-contained, discrete dosage form. Carbon nanotube patches were developed for the delivery of nicotine and were proved effective.^89,90^

Fullerenes are nanoparticles with spherical structure, specific geometry, and strong non-polar character which enables their use in lipid-like systems. The most common fullerene synthesized is the C60 which is produced at 1000°C.

Their antioxidant activity and the ability to interact with the epidermal keratinocytes makes them a suitable nanomaterial to be used in transdermal drug delivery. Due to their antioxidant activity, they are very popular for use in cosmetic products such as moisturizers, sunscreens, hair growth stimulators, etc.^91^

Fullerenes have various advantages including high biocompatibility and low cytotoxicity which makes them an excellent system for targeted drug delivery.^92^

Fullerenes migrate intracellularly when they come in contact with the skin and thus can be used to encapsulate an active compound that will be released into the epidermis after topical application. Inui et al formulated polyhydroxylated fullerenes for the treatment of acne vulgaris. They tested their formulation in-vitro on sebum production in hamster sebocytes and the results showed that fullerenes are a potent system for the treatment of acne vulgaris and are beneficial in skincare.^93^

## Selection of nano-carrier based on drug properties

Every nanocarrier system is not suitable for transportation of all kinds of drug molecules, therefore the suitable nanocarrier system is selected keeping in mind the physiochemical properties of the drug molecules.

Liposomes comprise of a lipid bilayer, thus they are suitable carriers for both lipophilic and hydrophilic drug molecules. The lipophilic molecules can be loaded between the bilayer and the hydrophilic molecules can be loaded inside the core. Some examples include melatonin, estradiol, etc. Transfersomes have similar structure to liposomes thus they can also transport both lipophilic and hydrophilic molecules like corticosteroids, ketoprofen, etc.

Ethosomes and niosomes are easy to prepare soft and efficient structures and are suitable for the transport of highly lipophilic drugs and some hydrophilic drugs too, like minoxidil, propranolol, ellagic acid, etc.

Lipid nanoparticles are suitable for the transport of both hydrophilic and hydrophobic drugs but have more preference for hydrophobic drugs like finasteride, minoxidil, etc.^94^

## Conclusion

Dermal and transdermal drug delivery is the next generation drug delivery system for its sustained, controlled release of both hydrophobic and hydrophilic drugs. Additionally, it has acquired significant potential, addressing the limited oral bioavailability of drugs and inconvenience of injections effectively. The biggest obstacle in the delivery of transdermal drugs is the barrier existence of the skin, which prevents most of the drugs from penetrating. Thus, to resolve the arising problem, various nanocarriers and vesicular systems are designed and developed to simplify the process of drug therapies These vesicle and particle systems have been characterized for their simplicity of preparation, safety, and efficacy, and incorporation of these in transdermal patches, creams and gels will improve the drug permeation through the skin.

## Ethical Issues

Not applicable

## Conflict of Interest

The author reports no conflicts of interest.
